# Polymorphisms in the FTO Gene and Their Association With Cancer Risk: A Comprehensive Review and Meta‐Analysis

**DOI:** 10.1002/cnr2.70162

**Published:** 2025-05-20

**Authors:** Fengran Guo, Yilong Gao, Hu Wang, Pengfei Zhou, Yanping Zhang, Zhihai Teng, Yaxuan Wang, Zhenwei Han

**Affiliations:** ^1^ Department of Urology The Second Hospital of Hebei Medical University Shijiazhuang China; ^2^ Department of Urology Institute of Urology, West China Hospital, Sichuan University Chengdu China; ^3^ Department of Urology The First Hospital of Jiaxing Zhejiang China; ^4^ Zhengding Country People's Hospital zhengding China

**Keywords:** cancer, FTO, meta‐analysis, polymorphism, SNP

## Abstract

**Background:**

This meta‐analysis aimed to clarify the connection between six polymorphisms in the FTO gene and susceptibility to cancer.

**Methods:**

The relevant literature on the relationship between FTO variants and cancer susceptibility was comprehensively gathered from PubMed, Scopus, Embase, Medline, and Web of Science prior to May 20, 2024.

**Results:**

Our analysis revealed that FTO rs9939609 had a certain correlation with an elevated cancer risk within the Asian demographic q vs. r (OR = 1.22, 95% CI = 1.07–1.39, *p* = 0.003); rq + qq vs. rr (OR = 1.18, 95% CI = 1.04–1.35, *p* = 0.011); qq vs. rr + rq (OR = 1.78, 95% CI = 1.39–2.27, *p* = 0.001). Additionally, FTO rs1477196 was linked to a higher risk of thyroid cancer qq vs. rr + rq (OR = 1.47, 95% CI = 1.13–1.91, *p* = 0.004) and remarkably relevant to an increased cancer susceptibility for Caucasians (q vs. r (OR = 1.29, 95% CI =1.06–1.57, *p* = 0.009); rq + qq vs. rr (OR = 1.37, 95% CI = 1.04–1.80, *p* = 0.024)). In the stratified analysis of rs8047395, the results indicated that rs8047395 had a certain correlation with cancer susceptibility for thyroid cancer q vs. r (OR = 1.23, 95% CI = 1.01–1.51, *p* = 0.041) and qq vs. rr + rq (OR = 1.53, 95% CI = 1.24–1.91, *p* < 0.01).

**Conclusion:**

FTO rs9939609 showed a correlation with cancer risk among individuals of Asian descent. FTO rs1477196 was correlated with an increased risk for thyroid cancer and remarkably relevant to an increased cancer susceptibility for Caucasians. FTO rs8047395 was associated with the risk of thyroid cancer.

**Abbreviations**
CI: confidence interval
DHPLC: denaturing high‐performance liquid chromatography
HB: hospital‐based
HWE: Hardy–Weinberg equilibrium
*n*
: number
N: no
NA: not applicable
OR: odds ratio
PB: population‐based
PCa: prostate cancer
PCR: polymerase chain reaction
SNP: single nucleotide polymorphism
Y: yes

## 1 Introduction

The Global Cancer Facts Report from the American Cancer Society indicates that cancer continues to be the primary cause of mortality in children across the United States. In North America, the typical 5‐year survival rates for various adult cancer types range from 14% to 56%, which remain considerably low [1]. At present, cancer ranks as the primary or secondary cause of early mortality in many nations globally, and the global incidence of cancer is projected to rise over the next 50 years [2]. Cancer inevitably leads to a huge expenditure on health, human global potential losses, and social and economic loss [3, 4]. Developing programs aimed at treating and preventing cancer is a huge challenge to advancing health today. Obesity serves as a risk factor for the onset of cancer and is linked to unfavorable outcomes in various tumor types. Investigating the relationship between obesity‐related genes and cancer has become crucial for improving cancer therapies [4].

FTO is the first gene associated with obesity susceptibility that was discovered through genome‐wide association studies (GWAS) [5]. Nonetheless, advancements in bioinformatics have suggested that FTO functions as a 2‐oxoglutarate (2‐OG) Fe(II)‐dependent demethylase, closely resembling the bacterial DNA demethylase AlkB and the mammalian homologs ABH1 and ABH2 [6]. It has been confirmed that FTO can regulate chromatin‐related RNA, especially the m6A modification of RNA [7]. This establishes a theoretical foundation for investigating how FTO gene polymorphisms influence cancer. Researching the effects of these polymorphisms on cancer offers guidance for enhancing our understanding of the disease and its treatment.

Hernández‐Caballero ME and Sierra‐Ramírez JA conducted a comprehensive review in 2015 on the single nucleotide polymorphisms (SNPs) of the FTO gene and their associations with cancer risk, laying a solid foundation for understanding the genetic factors involved. Figlioli's meta‐analysis provided an in‐depth evaluation of polymorphisms associated with differentiated thyroid cancer, enhancing our specific understanding of the risk factors for this type of cancer [8]. Gholamalizadeh's study focused on the relationship between FTO polymorphisms and colorectal cancer, thereby broadening the scope of research on FTO across different cancer types [9]. Huang's meta‐analysis examined the association between the FTO gene polymorphism rs9939609 and cancer risk, offering robust evidence from a large population and highlighting the widespread nature of this polymorphism across various cancers [10]. Li's meta‐analysis, involving 16,277 cases and 31,153 controls, further elucidated the relationship between FTO polymorphisms and cancer risk [11]. Zhao's meta‐analysis specifically explored the relationship between the FTO rs8050136 polymorphism and cancer risk, expanding our knowledge of specific FTO polymorphisms [12]. Finally, Zhu's meta‐analysis on FTO expression in gastric cancer investigated clinical pathological features and prognosis, emphasizing the broader role of FTO beyond genetic polymorphisms [13].

Although these studies have significantly advanced our understanding of the relationship between FTO gene polymorphisms and cancer risk, our research offers several distinct advantages. We evaluated a wider spectrum of polymorphisms and cancer types, thereby providing a more comprehensive overview of potential genetic associations. By incorporating the latest data and studies, our meta‐analysis reflects current research trends, enhancing the accuracy and relevance of our conclusions. Additionally, we conducted detailed subgroup analyses based on race and age, offering insights into population‐specific risks that were not fully explored in earlier studies. Furthermore, we included gender as an additional stratification factor, ensuring a more nuanced understanding of the genetic factors influencing cancer susceptibility.

Initially, a meta‐analysis of *FTO* gene polymorphism mainly focused on obesity. Recently, an increasing number of studies have identified the influence of FTO gene polymorphisms on cancer. However, these studies are inconsistent. Consequently, it is essential to perform a thorough meta‐analysis to assess how FTO gene polymorphisms affect cancer susceptibility.

## 2 Materials and Methods

### 2.1 Literature Search

We performed an extensive literature review using PubMed, Medline, Scopus, Embase, and Web of Science to explore the possible link between FTO polymorphism and cancer risk, employing the following search strategy: (FTO OR Fat mass and obesity‐associated) AND (polymorphism OR mutation OR variation OR SNP OR genotype) AND (cancer OR tumor OR neoplasm OR malignancy OR carcinoma OR adenocarcinoma). English was limited to the language of literature. After careful and meticulous screening, we identified six FTO polymorphisms (rs1121980, rs1477196, rs7206790, rs8047395, rs8050136, and rs9939609) for further study.

### 2.2 Inclusion Criteria and Exclusion Criteria

A set of inclusion criteria was used for our meta‐analysis [1]. Case–control research examining the association of FTO polymorphism with cancer risk [2]. Population genetic polymorphism publications only [3, 4]. Adequate genotype information that meets the requirements for estimating odds ratios (ORs) and their associated 95% confidence intervals [4]. The control participants were in accordance with Hardy–Weinberg equilibrium (HWE). To guarantee that the studies incorporated in this meta‐analysis were of high quality and directly aligned with our research goals, we established several exclusion criteria, such as case‐only studies, case reports, reviews, and studies lacking raw data.

### 2.3 Data Extraction

The data was independently extracted by (FG and HW) and all case–control studies met the inclusion criteria. Two researchers have reached a consensus on any controversy. The information extracted from the qualifying articles included the first author's name, publication year, ethnicity, control source, cancer type, and the number of cases and controls for FTO genotypes. In this study, races were divided into four categories: “Caucasian” “Asian” “African,” and “mixed race”. Cancer types were classified as “breast cancer”, “thyroid cancer,” and “other cancer.”

### 2.4 Statistical Analysis

Aggregating OR and corresponding 95% CI was a method for assessing the risk between FTO polymorphism and cancer in allelic (q vs. r), dominant (rq + qq vs. rr), and recessive qq vs. rr + rq models (r: wild allele; q: mutated allele). The Cochrane Q‐statistic and I^2^ statistic were employed to assess heterogeneity among the studies. The choice between using a random effects model or a fixed effects model was based on the level of heterogeneity. When heterogeneity was not significant (*I* ^2^ < 50% and *p* ≥ 0.1), fixed effects models were utilized. In contrast, the random effects model was employed when *I* ^2^ > 50% or *p* < 0.1. Subgroup meta‐analyses were conducted based on ethnicity and control source. To assess the robustness of the findings, sensitivity analyses were also performed. Hardy–Weinberg equilibrium (HWE) was evaluated using an asymptotic test, with deviations noted when *p* < 0.05. The potential for publication bias in the eligible studies was assessed quantitatively through Begg's and Egger's regression tests.

### 2.5 In Silico Analysis Using GTEx Website

To examine the impact of FTO polymorphisms, we assessed the relationship between these polymorphisms and FTO expression levels utilizing data from the GTEx cohort [14].

## 3 Result

### 3.1 The Key Features of This Study

**FIGURE 1:**
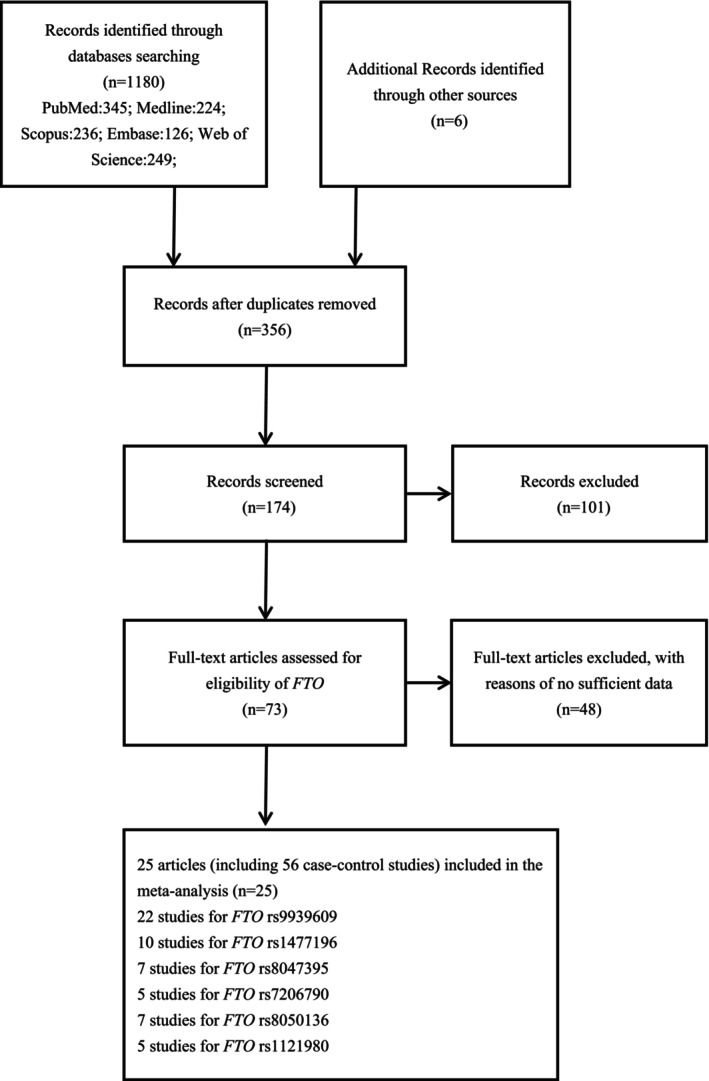
Flow chart of studies selection process for *FTO* gene polymorphisms.

The process of study selection is illustrated in Figure 1. Our meta‐analysis included a total of 25 articles, which comprised 56 case–control studies [15, 16, 17, 18, 19, 20, 21, 22, 23, 24, 25, 26, 27, 28, 29, 30, 31, 32, 33, 34, 35]. Altogether, there were 23,880 cases and 36,262 controls, including six polymorphisms of the FTO gene (rs9939609, rs1121980, rs1477196, rs7206790, rs8047395, rs8050136) met the inclusion criteria. Six studies were population‐based, 49 were hospital‐based, and one study was population‐based and hospital‐based. Table 1 presents the characteristics of all eligible studies along with the genotype frequency distribution for the six FTO polymorphisms included in our meta‐analysis. The quality of the selected studies was assessed using the Newcastle‐Ottawa Scale (NOS), as detailed in Table S1.

**TABLE 1:** Characteristics of eligible case–control studies included in the meta‐analysis.

SNP	First author	Year	Ethnicity	Source of control	Cancer type	Case			Control			HWE
						AA	AB	BB	AA	AB	BB	
rs9939609	da Cunha	2013	Caucasian	HB	BC	37	50	13	48	78	22	Y
	Lurie	2011	Caucasian	HB/PB	OC	1236	1662	663	1856	2463	848	Y
	Tang	2011	Mixed	HB	OC	386	493	174	436	527	167	Y
	Kaklamani	2011	Mixed	HB	BC	129	124	49	136	161	52	Y
	Tarabra	2012	Caucasian	HB	OC	96	182	63	94	154	63	Y
	Kitahara	2012	Caucasian	HB	TC	144	155	41	151	220	72	Y
	Lin	2013	Asian	HB	OC	213	133	14	271	116	13	Y
	Mojaver	2015	Caucasian	HB	BC	26	25	11	20	32	10	Y
	Zeng	2015	Asian	HB	BC	389	130	17	408	119	10	Y
	Nock(C)	2011	Caucasian	HB	OC	60	99	25	190	286	97	Y
	Nock(A)	2011	African	HB	OC	42	61	34	76	177	77	Y
	Yamaji	2020	Asian	PB	OC	212	111	20	448	191	27	Y
	He	2021	Asian	HB	OC	129	33	9	175	51	2	Y
	Hoang	2021	Asian	HB	TC	534	159	12	525	171	9	Y
	Hua	2021	Asian	HB	OC	310	81	11	914	262	22	Y
	Khella	2018	Egyptian	PB	OC	15	54	17	52	76	21	Y
	Li	2022	Asian	HB	OC	302	102	6	420	129	5	Y
	Liao	2021	Asian	HB	OC	236	68	10	294	80	6	Y
	Moshtaghioun	2021	Caucasian	HB	TC	33	31	16	30	35	15	Y
	Thompson	2009	Mixed	HB	OC	90	252	217	121	336	261	Y
	Doaei	2022	Asian	HB	BC	56	104	20	126	202	32	Y
	Mansoor	2023	Asian	PB	BC	98	277	275	52	105	43	Y
rs1477196	Kaklamani	2011	Mixed	HB	BC	123	130	58	164	154	40	Y
	Kitahara	2012	Caucasian	HB	TC	115	164	60	182	203	54	Y
	Mojaver	2015	Caucasian	HB	BC	33	25	4	37	20	5	Y
	Zeng	2015	Asian	HB	BC	272	231	33	231	254	52	Y
	Fan	2022	Asian	HB	OC	203	121	28	834	569	107	Y
	He	2021	Asian	HB	OC	105	58	8	127	87	14	Y
	Hoang	2021	Asian	HB	TC	333	291	81	340	306	59	Y
	Hua	2021	Asian	HB	OC	220	145	37	643	462	93	Y
	Liao	2021	Asian	HB	OC	178	117	19	216	138	26	Y
	Akilzhanova	2013	Mixed	HB	BC	126	156	33	252	266	86	Y
rs8047395	Kaklamani	2011	Mixed	HB	BC	89	140	80	101	172	82	Y
	Kitahara	2012	Caucasian	HB	TC	71	170	100	120	234	86	Y
	Fan	2022	Asian	HB	OC	143	147	62	595	701	214	Y
	He	2021	Asian	HB	OC	75	78	18	75	116	37	Y
	Hoang	2021	Asian	HB	TC	249	326	130	256	352	97	Y
	Hua	2021	Asian	HB	OC	158	161	83	450	557	191	Y
	Liao	2021	Asian	HB	OC	124	151	39	142	182	56	Y
rs7206790	Kaklamani	2011	Mixed	HB	BC	102	142	71	121	163	74	Y
	Fan	2022	Asian	HB	OC	263	81	8	1085	382	43	Y
	He	2021	Asian	HB	OC	110	53	8	165	59	4	Y
	Hua	2021	Asian	HB	OC	308	80	14	892	275	31	Y
	Liao	2021	Asian	HB	OC	214	88	12	275	96	9	Y
rs8050136	Tang	2011	Mixed	HB	OC	375	504	176	428	533	166	Y
	Kitahara	2012	Caucasian	HB	TC	144	156	41	150	222	72	Y
	Nock(C)	2011	Caucasian	HB	OC	59	101	24	189	286	97	Y
	Nock(A)	2011	African	HB	OC	52	57	28	93	171	65	Y
	Yamaji	2020	Asian	PB	OC	212	112	20	449	191	27	Y
	Hoang	2021	Asian	HB	TC	536	157	12	526	170	9	Y
	Thompson	2009	Mixed	HB	OC	91	250	217	119	336	265	Y
rs1121980	da Cunha	2013	Caucasian	HB	BC	37	47	16	46	70	32	Y
	Kitahara	2012	Caucasian	HB	TC	136	155	49	137	223	82	Y
	Zeng	2015	Asian	HB	BC	360	154	22	377	145	15	Y
	Yamaji	2020	Asian	PB	OC	197	124	23	417	213	37	Y
	Hoang	2021	Asian	HB	TC	493	196	16	482	207	16	Y

Abbreviations: A, asian; BC, breast cancer; C, caucasian; central nervous system tumor; colorectal adenoma; endometrial cancer; gastric cancer; glioma, pancreatic cancer; HB, hospital‐based; HB/PB, hospital‐based and population‐based; hepatoblastoma; HWE; hardy–weinberg equilibrium; malignant pleural Mesothelioma; OC, wilms tumor; PB, population‐based; SNP, single nucleic polymorphism; TC, thyroid cancer; Y, yes.

### 3.2 Quantitative Evaluation

#### 3.2.1 rs9939609

**FIGURE 2:**
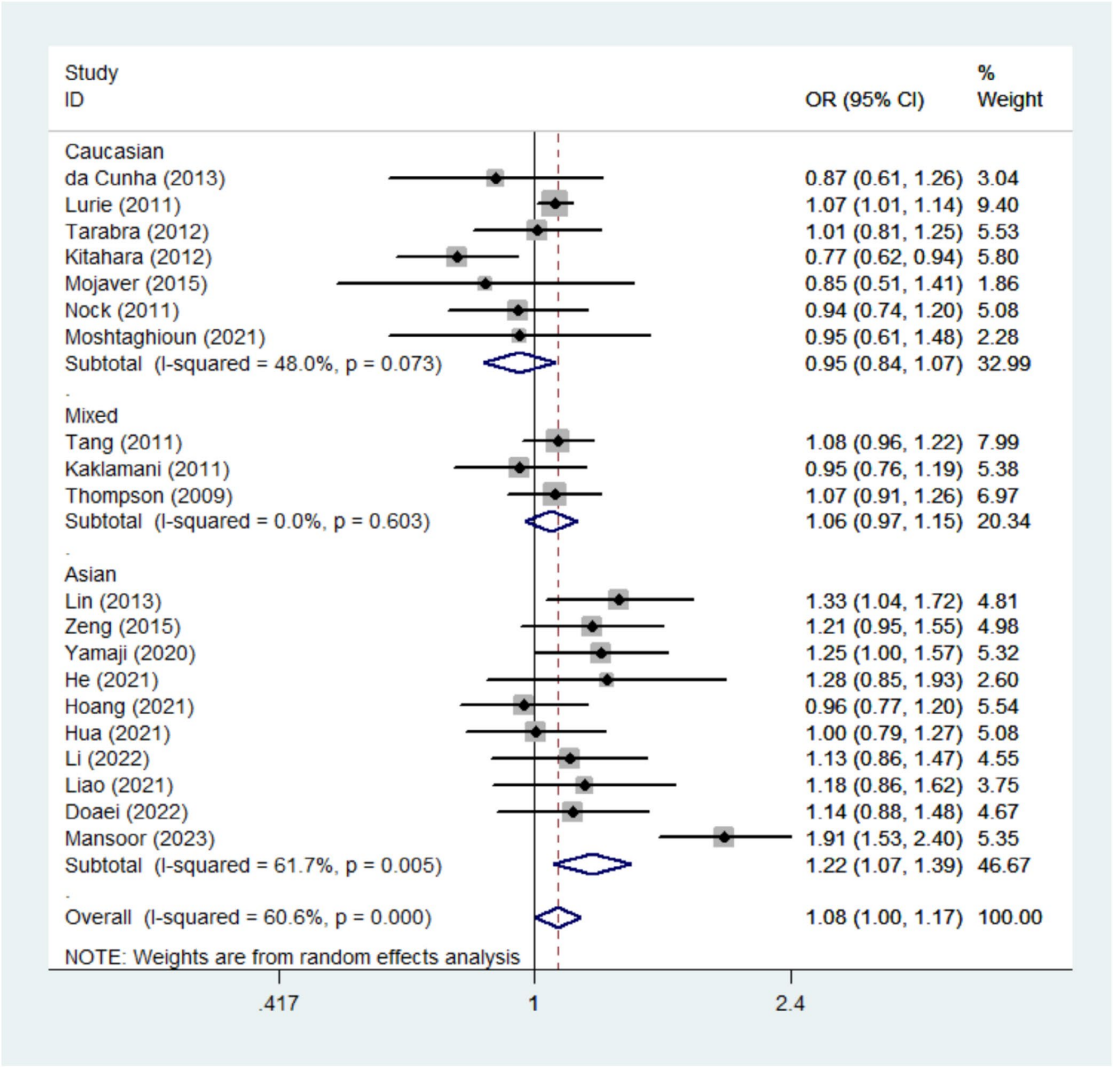
Forest plot of *FTO* rs9939609 polymorphism and cancer risk in allele contrast model stratified by ethnicity.

A meta‐analysis was performed involving 22 studies (including 10 876 cases and 14 688 controls) of rs9939609, and the findings indicated a significant correlation between rs9939609 and cancer risk in allele contrast (OR = 1.09, 95% CI = 1.01–1.17, *p* = 0.031) and recessive model (OR = 1.20, 95% CI = 1.05–1.38, *p* = 0.010). In the stratified analysis based on cancer type, the results suggested that rs9939609 was associated with an increased risk of other cancers (allele contrast (OR = 1.09, 95% CI = 1.03–1.15, *p* = 0.002), Dominant model (OR = 1.10, 95% CI = 1.01–1.21, *p* = 0.037), recessive model (OR = 1.15, 95% CI = 1.05–1.26, *p* = 0.001)). Nevertheless, in the stratified analysis by ethnicity, we found that the rs9939609 polymorphism was significantly associated with cancer susceptibility among Asians (OR = 1.22, 95% CI = 1.07–1.39, *p* = 0.003); Dominant model (OR = 1.18, 95% CI = 1.04–1.35, *p* = 0.011); Recessive model (OR = 1.78, 95% CI = 1.39–2.27, *p* = 0.001). (Figure 2, Table 2).

**TABLE 2:** Meta‐analysis of rs9939609.

Variables	*n*	Allele contrast		Dominant model		Recessive model	
		*p*, OR (99% CI)	*p* (Q test), *I* ^*2*^	*p*, OR (99% CI)	*p* (Q test), *I* ^*2*^	*p*, OR (99% CI)	*p* (Q test), *I* ^*2*^
Total	22	**0.031**, 1.09 (1.01, 1.17)	0.00, 61.3%	0.162, 1.07 (0.97, 1.17)	0.004, 50.7%	**0.010**, 1.20 (1.05, 1.38)	0.009, 46.7%
Cancer type							
BC	6	0.327, 1.14 (0.88, 1.47)	0.000, 80.2%	0.519, 1.10 (0.83, 1.45)	0.010, 66.7%	0.092, 1.42 (0.94, 2.13)	0.014, 64.7%
TC	3	0.081, 0.87 (0.74, 1.02)	0.302, 16.5%	0.057, 0.83 (0.69, 1.01)	0.341, 7.1%	0.408, 0.86 (0.60, 1.23)	0.336, 8.3%
OC	13	**0.002**, 1.09 (1.03, 1.15)	0.336, 10.9%	**0.037**, 1.10 (1.01, 1.21)	0.138, 30.8%	**0.004**, 1.15 (1.05, 1.26)	0.409, 3.8%
Ethnicity							
Asian	11	**0.003**, 1.22 (1.07, 1.39)	0.005, 61.7%	**0.011**, 1.18 (1.04, 1.35)	0.079, 41.7%	**0.001**, 1.78 (1.39, 2.27)	0.313, 14.1%
Caucasian	7	0.389, 0.95 (0.84, 1.07)	0.073, 48.0%	0.436, 0.94 (0.81, 1.10)	0.160, 35.1%	0.133, 0.96 (0.79, 1.17)	0.170, 33.8%
Mixed	3	0.234, 1.06 (0.97, 1.15)	0.603, 0.0%	0.640, 1.03 (0.90, 1.18)	0.425, 0.00%	0.671, 1.12 (0.97, 1.31)	0.984, 0.0%

Abbreviations: BC, breast cancer; CI, confidence interval; *n*, number; OC, other cancer; OR, odds ratio; TC, thyroid cancer.

#### 3.2.2 rs1121980

Five studies, comprising 2025 cases and 2499 controls, were incorporated into this meta‐analysis to explore the relationship between rs1121980 and cancer susceptibility. The stratified analysis by ethnicity revealed that rs1121980 was negatively correlated with cancer susceptibility among Caucasians in allele contrast (OR = 0.77, 95% CI = 0.64–0.92, *p* = 0.004) and Dominant model (OR = 0.69, 95% CI = 0.54–0.90, *p* = 0.006) (Table 3).

**TABLE 3:** Meta‐analysis of rs1121980.

Variables	*n*	Allele contrast		Dominant model		Recessive model	
		*p*, OR (99% CI)	*p* (Q test), *I* ^*2*^	*p*, OR (99% CI)	*p* (Q test), *I* ^*2*^	*p*, OR (99% CI)	*p* (Q test), *I* ^*2*^
Total	5	0.708, 0.97 (0.80, 1.16)	0.012, 68.9%	0.673, 0.95 (0.76, 1.19)	0.019, 66.1%	0.521, 0.92 (0.72, 1.17)	0.281, 21.0%
Cancer type							
BC	2	0.939, 1.01 (0.70, 1.48)	0.075, 68.4%	0.962, 1.01 (0.70, 1.46)	0.181, 44.2%	0.970, 1.01 (0.64, 1.60)	0.109, 61.0%
TC	2	0.132, 1.18 (0.95, 1.46)	0.136, 55.0%	0.171, 0.80 (0.59, 1.10)	0.091, 65.1%	0.176, 0.79 (0.57, 1.11)	0.456, 0.0%
Ethnicity							
Asian	3	0.269, 1.09 (0.94, 1.27)	0.220, 33.9%	0.350, 1.09 (0.91, 1.30)	0.219, 34.1%	0.261, 1.23 (0.86, 1.76)	0.072, 0.0%
Caucasian	2	**0.004**, 0.77 (0.64, 0.92)	0.864, 0.0%	**0.006**, 0.69 (0.54, 0.90)	0.675, 0.0%	0.060, 0.73 (0.52, 1.01)	0.862, 0.0%

Abbreviations: BC, breast cancer; CI, confidence interval; *n*, number; OC, other cancer; OR, odds ratio; TC, thyroid cancer.

#### 3.2.3 rs1477196

**FIGURE 3:**
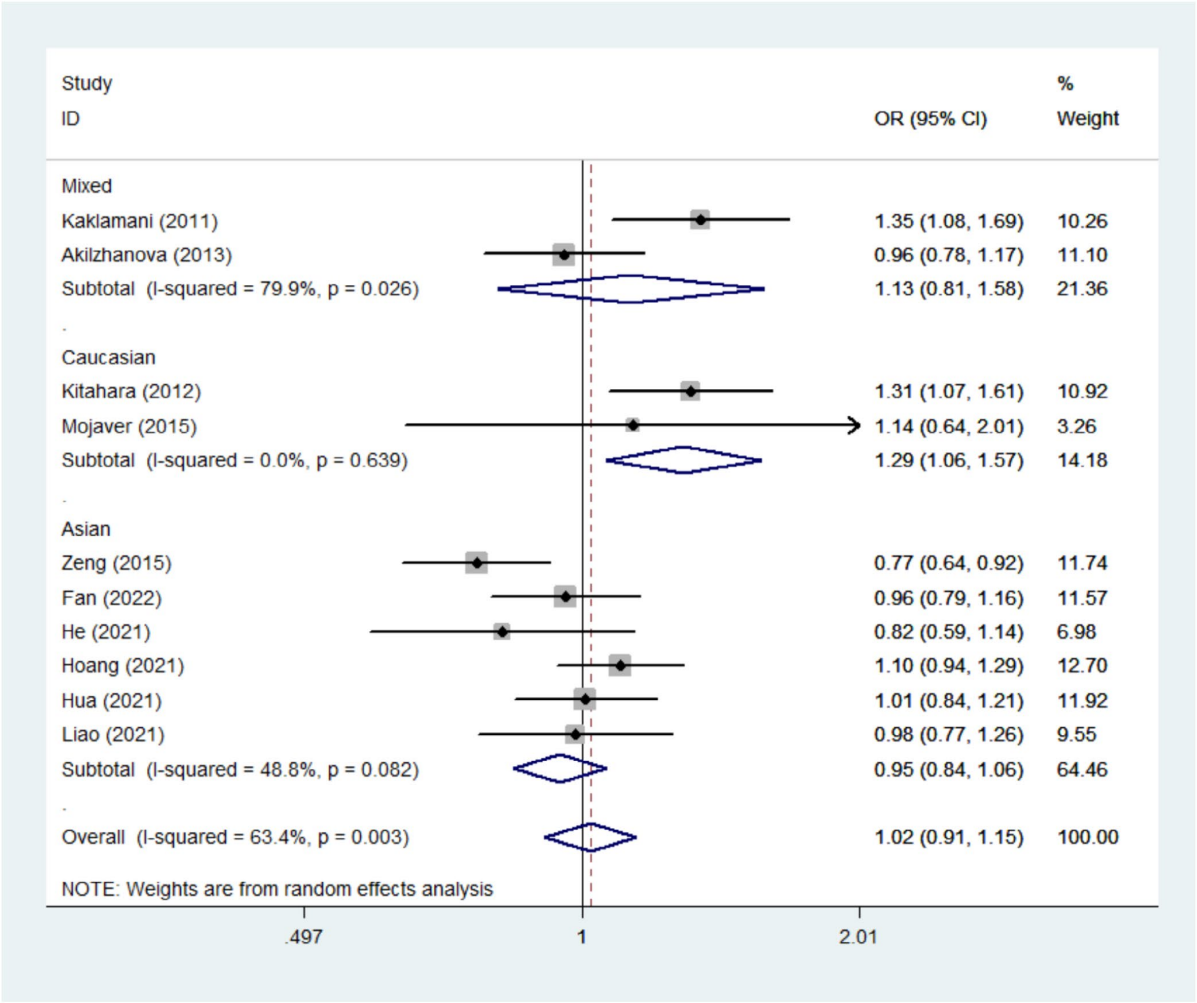
Forest plot of *FTO* rs1477196 polymorphism and cancer risk in allele contrast model stratified by ethnicity.

Ten studies, which included 3507 cases and 6021 controls, were utilized in the meta‐analysis to examine the relationship between rs1477196 and cancer susceptibility. In the stratification analysis by cancer type, the results indicated that rs1477196 had a certain correlation with cancer susceptibility for thyroid cancer in the recessive model (OR = 1.47, 95% CI = 1.13–1.91, *p* = 0.004). Additionally, the subgroup analysis based on ethnicity showed that rs1477196 was significantly associated with cancer susceptibility among Caucasians (allele contrast (OR = 1.29, 95% CI =1.06–1.57, *p* = 0.009); Dominant model (OR = 1.37, 95% CI = 1.04–1.80, *p* = 0.024)). (Figure 3, Table 4).

**TABLE 4:** Meta‐analysis of rs1477196.

Variables	*n*	Allele contrast		Dominant model		Recessive model	
		*p*, OR (99% CI)	*p* (Q test), *I* ^*2*^	*p*, OR (99% CI)	*p* (Q test), *I* ^*2*^	*p*, OR (99% CI)	*p* (Q test), *I* ^*2*^
Total	9	0.652, 1.03 (0.91, 1.17)	0.002, 66.8%	0.985, 1.00 (0.87, 1.15)	0.037, 51.2%	0.260, 1.15 (0.90, 1.46)	0.032, 52.5%
Cancer type							
BC	3	0.948, 1.01 (0.77, 1.33)	0.002, 79.8%	0.907, 1.03 (0.65, 1.61)	0.011, 77.8%	0.990, 0.99 (0.42, 2.35)	0.003, 83.0%
TC	2	0.050, 1.19 (1.00, 1.41)	0.182, 43.7%	0.249, 1.17 (0.89, 1.54)	0.126, 57.3%	**0.004**, 1.47 (1.13, 1.91)	0.780, 0.0%
OC	4	0.531, 0.97 (0.87, 1.08)	0.751, 0.0%	0.289, 0.93 (0.81, 1.06)	0.801, 0.0%	0.577, 1.07 (0.83, 1.39)	0.701, 0.0%
Ethnicity							
Asian	6	0.350, 0.95 (0.84, 1.06)	0.082, 48.8%	0.109, 0.91 (0.82, 1.02)	0.327, 13.6%	0.923, 1.01 (0.77, 1.33)	0.084, 48.5%
Caucasian	2	**0.009**, 1.29 (1.06, 1.57)	0.639, 0.00%	**0.024**, 1.37 (1.04, 1.80)	0.881, 0.0%	0.055, 1.45 (0.99, 2.13)	0.357, 0.0%
Mixed	2	0.472, 1.13 (0.81,1.58)	0.026, 79.9%	0.143, 1.71 (0.95, 1.43)	0.382, 0.0%	0.794, 1.13 (0.45, 2.87)	0.002, 89.3%

Abbreviations: BC, breast cancer; CI, confidence interval; n, number; OC, other cancer; OR, odds ratio; TC, thyroid cancer.

#### 3.2.4 rs7206790

A meta‐analysis was performed involving 5 studies, which included 1554 cases and 3674 controls, to investigate the relationship between rs7206790 and cancer susceptibility. Nonetheless, no statistically significant association was found between the rs7206790 polymorphism and cancer predisposition (Figure S1).

#### 3.2.5 rs8047395

**FIGURE 4:**
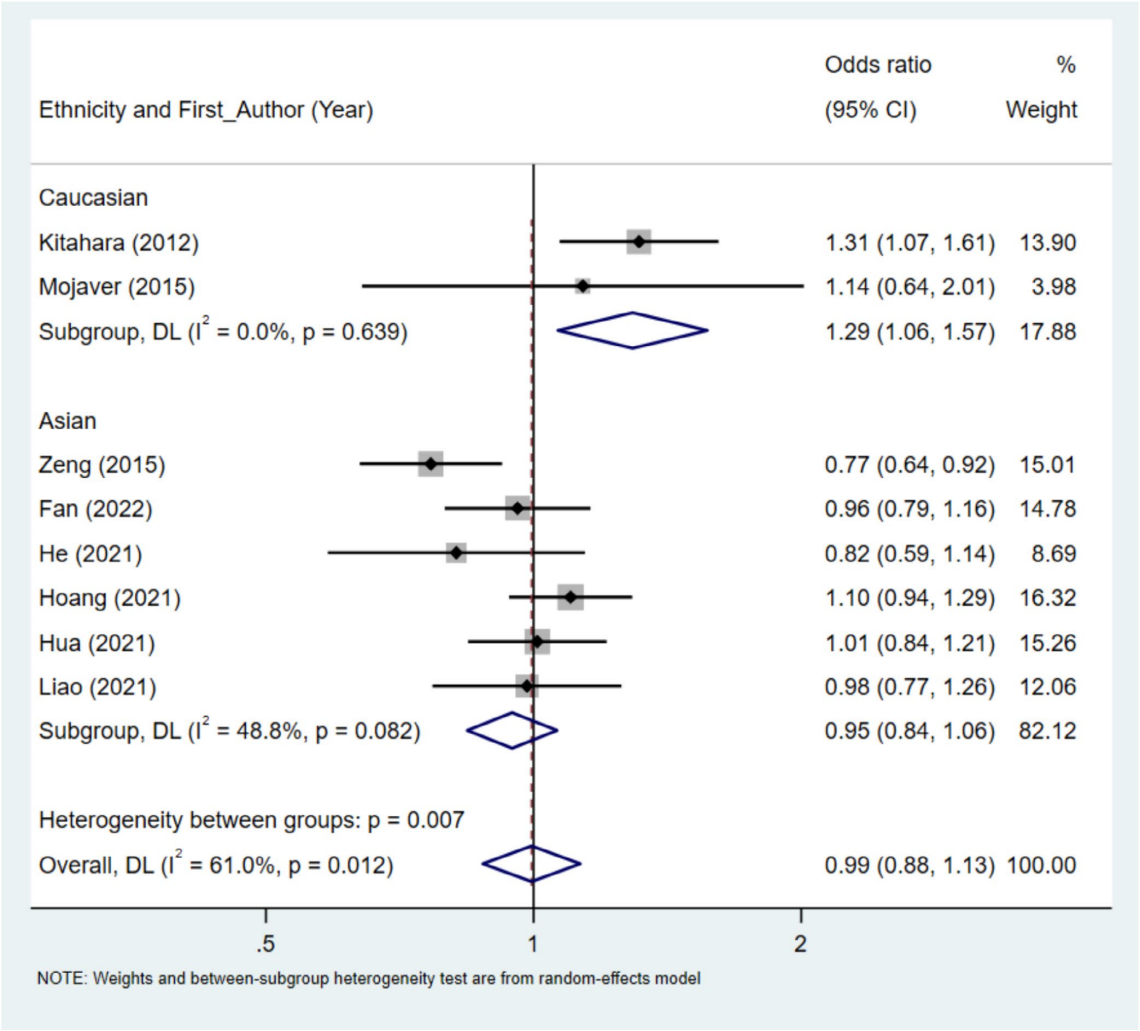
Forest plot of *FTO* rs8047395 polymorphism and cancer risk in allele contrast model stratified by cancer type.

Seven studies (including 2594 cases and 4816 controls) were analyzed in a meta‐analysis to explore the relationship between rs8047395 and cancer susceptibility. In the stratification analysis by cancer type, the results indicated that rs8047395 had a certain correlation with cancer predisposition for thyroid cancer in allele contrast (OR = 1.23, 95% CI = 1.01–1.51, *p* = 0.041) and recessive model (OR = 1.53, 95% CI = 1.24–1.91, *p* < 0.01). (Figure 4, Table 5).

**TABLE 5:** Meta‐analysis of rs8047395.

Variables	*n*	Allele contrast		Dominant model		Recessive model	
		*p*, OR (99% CI)	*p* (Q test), *I* ^*2*^	*p*, OR (99% CI)	*p* (Q test), *I* ^*2*^	*p*, OR (99% CI)	*p* (Q test), *I* ^*2*^
Total	7	0.506, 1.04 (0.92, 1.18)	0.009, 64.9%	0.679, 0.98 (0.88, 1.09)	0.118, 40.9%	0.055, 1.22 (1.00, 1.49)	0.033, 56.3%
Cancer type							
TC	2	**0.041**, 1.23 (1.01, 1.51)	0.105, 61.8%	0.141, 1.15 (0.96, 1.38)	0.126, 57.2%	**0.000**, 1.53 (1.24, 1.91)	0.404, 0.0%
OC	4	0.512, 0.95 (0.81, 1.11)	0.068, 58.0%	0.105, 0.89 (0.78, 1.02)	0.354, 7.9%	0.801, 1.04 (0.75, 1.45)	0.035, 65.2%
Ethnicity							
Asian	5	0.890, 0.99 (0.87, 1.13)	0.051, 57.6%	0.245, 0.93 (0.83, 1.05)	0.322, 14.5%	0.333, 1.13 (0.88, 1.47)	0.038, 60.5%

Abbreviations: BC, breast cancer; CI, confidence interval; *n*, number; OC, other cancer; OR, odds ratio; TC, thyroid cancer.

#### 3.2.6 rs8050136

A meta‐analysis was performed involving 7 studies (including 3324 cases and 4564 controls) of rs8050136 for examining the association between rs8050136 and cancer predisposition Nevertheless, no statistically significant association was observed between the rs8050136 polymorphism and cancer predisposition (Figure S2).

### 3.3 Evaluation of Sensitivity and Assessment of Publication Bias

A sensitivity analysis was carried out to examine the effect of individual studies on overall results, suggesting that the combined odds ratio for the six polymorphisms remained largely consistent (Figures [Link], [Link]). Additionally, Begg's and Egger's regression analyses were utilized to examine publication bias, indicating that bias was detected for rs8047395 in the recessive model and rs7206790 in the dominant model (*p* < 0.05) (Table S2).

### 3.4 In Silico Analysis Was Conducted Utilizing the GTEx Database

**FIGURE 5:**
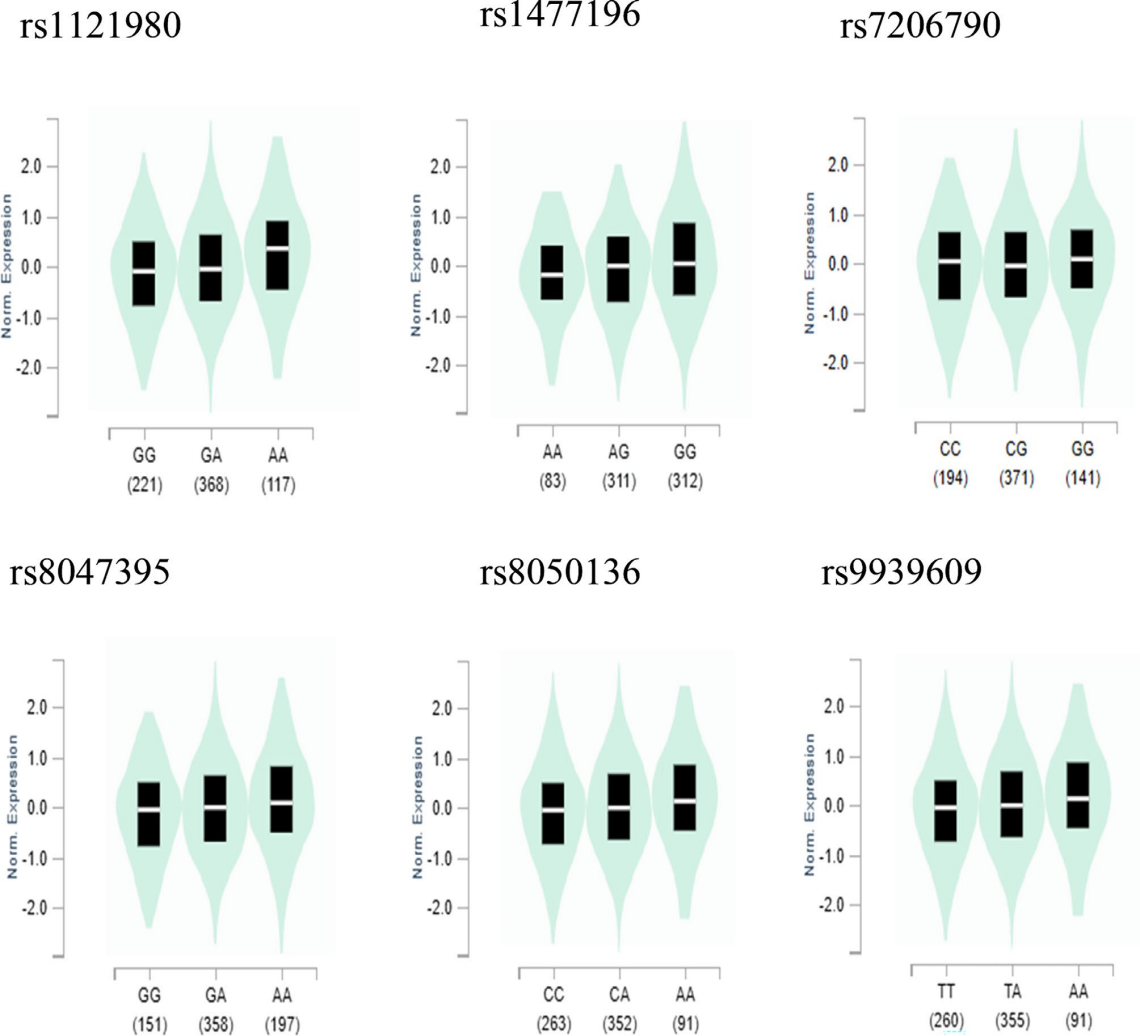
FTO mRNA expression by eQTL analysis in human tissues based on GTEx database. (rs1121980, rs1477196, rs7206790, rs8047395, rs8050136, rs9939609 detected in muscle‐skeletal).

According to the findings from the GTEx database, we noted that the mutant allele elevated FTO mRNA expression in rs1121980 (*p* = 1.7 × 10^−9^), rs1477196 (*p* = 2.9 × 10^−12^), rs7206790(*p* = 1.3 × 10^−4^), rs8047395(*p* = 2.2 × 10^−7^), rs8050136(*p* = 2.8 × 10^−6^), and rs9939609 (*p* = 4.2 × 10^−6^). (Figure 5).

## 4 Discussion

The FTO functions as an RNA N6‐methyladenosine (m6A) demethylase. Variants within the first intron of FTO are in strong linkage disequilibrium (LD) [36]. GWAS have shown that SNPs within FTO are significantly associated with obesity and cancer, while various investigations have highlighted the relationship between FTO's demethylase activity and cancer susceptibility at a molecular level [37]. Epidemiological research has indicated that SNPs in FTO are linked to an increased susceptibility to various cancers, including endometrial cancer [10], breast cancer [22], pancreatic cancer [32], and melanoma [38].

Earlier research has indicated that the FTO rs9939609 polymorphism serves as a risk factor for cancer among Asian populations [10, 11]. Our findings align with those of our meta‐analysis. Regarding FTO rs9939609, the analysis revealed that this polymorphism significantly heightened tumor susceptibility in both allelic and recessive models, with no evidence of publication bias detected. In a subgroup analysis categorized by ethnicity, we observed that rs9939609 notably increased cancer susceptibility among Asians across allelic comparisons as well as dominant and recessive models. This suggests that rs9939609 may elevate tumor risk in Asian populations; however, further studies with larger sample sizes are necessary for validation. Additionally, Huang's research indicated a potential association between the rs9939609 polymorphism and the susceptibility to endometrial and pancreatic cancers [10].

We found that not many studies have been reported on FTO rs1121980, especially its impact on cancer. Figlioli's research indicated that the FTO rs1121980 polymorphism is linked to an increased risk of differentiated thyroid carcinom [8]. Our analysis revealed that FTO rs1121980 did not show a significant association with cancer. When we conducted a subgroup analysis based on race, the findings indicated an inverse relationship between FTO rs1121980 and cancer susceptibility in Caucasians. This might be due to the inconsistent racial content of the samples. More large sample studies are needed to confirm this result.

No correlation was found between FTO rs8050136 and cancer susceptibility. Nonetheless, Zhao's research indicated that the FTO rs8050136 polymorphism might be linked to thyroid papillary carcinoma [12]. Which might be caused by insufficient sample size.

Our meta‐analysis revealed that FTO rs1477196 and rs8047395 were not significantly associated with cancer risk. However, we observed considerable heterogeneity within the analysis. A publication bias for rs8047395 was identified in the recessive model. To investigate the sources of this heterogeneity, we conducted a subgroup analysis. This analysis indicated that rs8047395 had a significant correlation with thyroid cancer risk. Additionally, it showed that FTO rs1477196 was notably related to susceptibility to thyroid cancer and an increased risk among Caucasians. However, FTO rs7206790 was found to have no correlation with cancer susceptibility. Kaklamani's research indicated that the polymorphisms rs1477196, rs8047395, and rs7206790 might be linked to susceptibility to breast cancer, with rs1477196 demonstrating the most significant association [22]. We posit that this may be attributed to our incorporation of both new and additional cases. More large sample studies are needed to confirm this result.

In this research, we performed a systematic and thorough search to achieve accurate and dependable results. The quality of the included studies was assessed using the NOS. To enhance the control over study quality, sensitivity analysis along with Begg's and Egger's regression tests was applied quantitatively. Nonetheless, this meta‐analysis has several limitations. First, Obesity has been widely recognized as an important risk factor for cancer progression. Yao et al.'s research also indicates that there is a genetic association between the FTO gene and both cancer and obesity [39]. However, this study did not explore the impact of FTO on cancer based on body mass index. Therefore, further research on the genetic relationship between FTO, obesity, and cancer is particularly important. Future large‐scale case–control studies will be necessary to more comprehensively reveal the mechanisms in this field. Second, the relatively small sample size restricts the reliability of our findings. Third, we only considered studies published in English, which may influence the effects of polymorphism. Fourth, most studies focused solely on the Han Chinese population. Fifth, we were unable to gather enough data to assess the relationship between FTO polymorphisms and more specific cancer types. Lastly, LD among FTO polymorphisms could impact FTO gene expression. Consequently, further large‐scale case–control studies are necessary to investigate how FTO gene polymorphisms affect cancer susceptibility.

## 5 Conclusion

This meta‐analysis indicates that FTO rs9939609 is linked to cancer susceptibility in the Asian population, while FTO rs1477196 is significantly relevant to thyroid cancer and an increased cancer susceptibility for Caucasians. FTO rs8047395 was remarkably associated with the risk of thyroid cancer. The FTO gene plays a significant role in the progression of cancer. Additional rigorously designed case–control studies are required to validate these results.

## Author Contributions

Z.H. had complete access to all the data in this study and is accountable for the integrity of the data as well as the accuracy of its analysis. Conceptualization and design of the study: F.G., H.W. Data acquisition: F.G., H.W. Data analysis and interpretation: Z.H., Y.Z., Manuscript preparation: F.G. Thorough revision of the manuscript for significant intellectual contributions: Z.T., Y.W. Statistical evaluation: H.W., Y.Z. No administrative, technical, or material support was provided.

## Ethics Statement

The authors have nothing to report.

## Conflicts of Interest

The authors declare no conflicts of interest.

## Supporting information


**Figure S1.** Forest plot of *FTO* rs7206790 polymorphism and cancer risk in allele contrast model.

**Figure S2.** Forest plot of FTO rs8050136 polymorphism and cancer risk in allele contrast model.

**Figure S3.** Sensitivity analysis of *FTO* rs9939609 (allelic comparison B vs. A).

**Figure S4.** Sensitivity analysis of *FTO* rs1121980 (allelic comparison B vs. A).

**Figure S5.** Sensitivity analysis of *FTO* rs1477196 (allelic comparison B vs. A).

**Figure S6.** Sensitivity analysis of *FTO* rs7206790 (allelic comparison B vs. A).

**Figure S7.** Sensitivity analysis of *FTO* rs8047395 (allelic comparison B vs. A).

**Figure S8.** Sensitivity analysis of *FTO* rs8050136 (allelic comparison B vs. A).

**Figure S9.** Funnel plot of *FTO* rs9939609 polymorphism (allelic comparison B vs. A).

**Figure S10.** Funnel plot of *FTO* rs1121980 polymorphism (allelic comparison B vs. A).

**Figure S11.** Funnel plot of *FTO* rs1477196 polymorphism (allelic comparison B vs. A).

**Figure S12.** Funnel plot of *FTO* rs7206790 polymorphism (allelic comparison B vs. A).

**Figure S13.** Funnel plot of *FTO* rs8047395 polymorphism (allelic comparison B vs. A).

**Figure S14.** Funnel plot of *FTO* rs8050136 polymorphism (allelic comparison B vs. A).

**Figure S15.** Performed an extensive literature review using PubMed, Medline, Scopus, Embase, and Web of Science.

**Table S1.** Methodological quality of the enrolled studies according to the Newcastle‐Ottawa scale.

**Table S2.** Publication bias of the six polymorphisms of *FTO*.

## Data Availability

The data that supports the findings of this study are available in the Supporting Information of this article.
