# Supplementary material for: Polymorphisms in the FTO Gene and Their Association With Cancer Risk: A Comprehensive Review and Meta‐Analysis
Source: Cancer Rep (Hoboken). 2025 May 20;8(5):e70162. doi: 10.1002/cnr2.70162 (PMC12089991; doi:10.1002/cnr2.70162)

**Supplementary figure 1.** Forest plot of *FTO* rs7206790 polymorphism and cancer risk in allele contrast model.


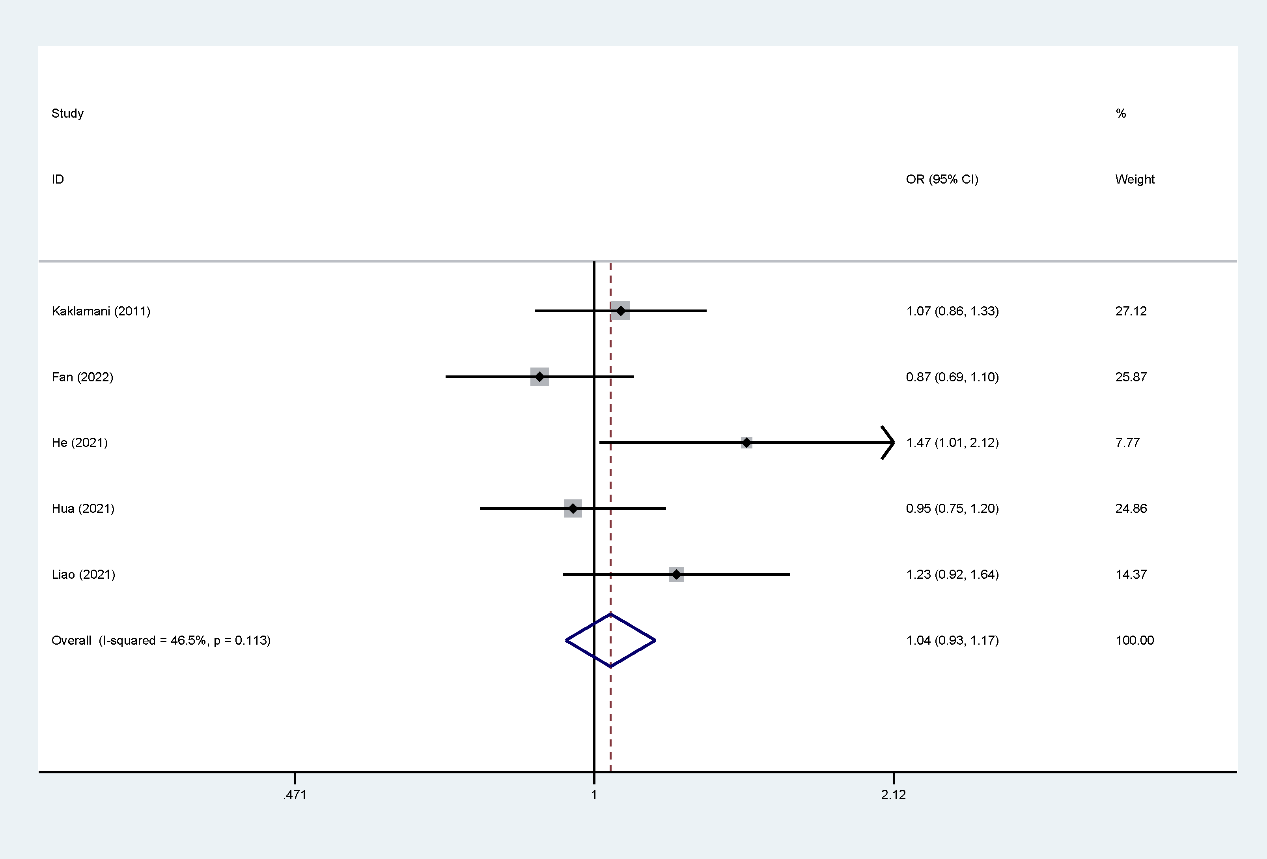

Supplement: Supplementary file 1 — Figure S1. Forest plot of FTO rs7206790 polymorphism and cancer risk in allele contrast model. [file CNR2-8-e70162-s012.docx]
