# Supplementary material for: Polymorphisms in the FTO Gene and Their Association With Cancer Risk: A Comprehensive Review and Meta‐Analysis
Source: Cancer Rep (Hoboken). 2025 May 20;8(5):e70162. doi: 10.1002/cnr2.70162 (PMC12089991; doi:10.1002/cnr2.70162)

**Supplementary figure 2.** Forest plot of FTO rs8050136 polymorphism and cancer risk in allele contrast model.


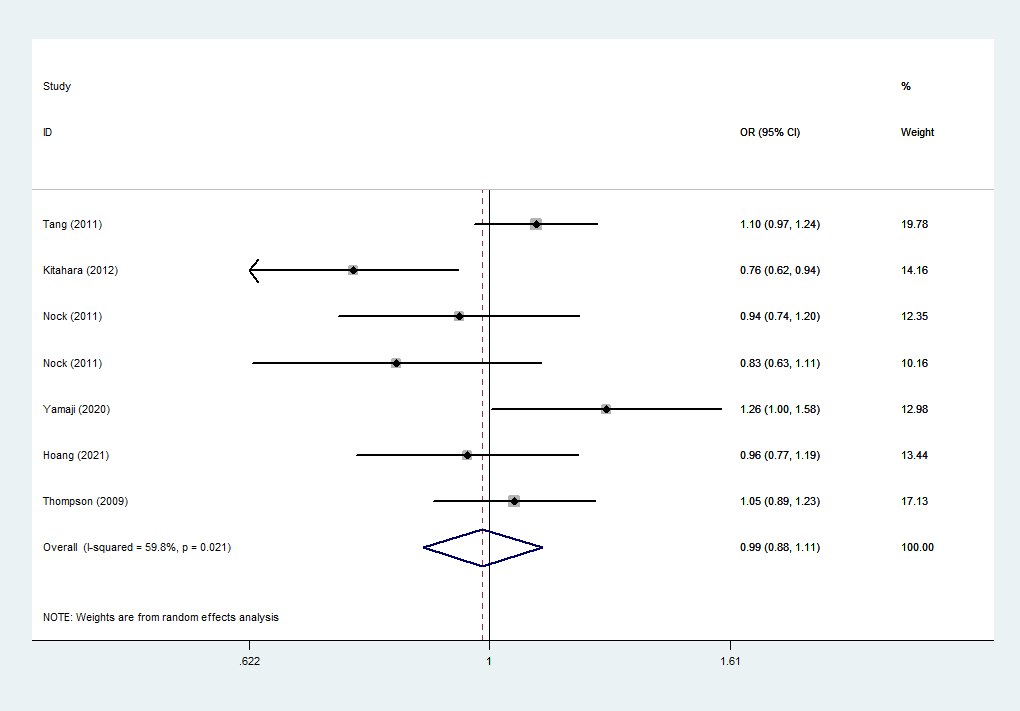

Supplement: Supplementary file 2 — Figure S2. Forest plot of FTO rs8050136 polymorphism and cancer risk in allele contrast model. [file CNR2-8-e70162-s005.docx]
