# Supplementary figures and images for: Polymorphisms in the FTO Gene and Their Association With Cancer Risk: A Comprehensive Review and Meta‐Analysis
Source: Cancer Rep (Hoboken). 2025 May 20;8(5):e70162. doi: 10.1002/cnr2.70162 (PMC12089991; doi:10.1002/cnr2.70162)

**Supplementary figure 3.** Sensitivity analysis of *FTO* rs9939609 (allelic comparison B vs. A).


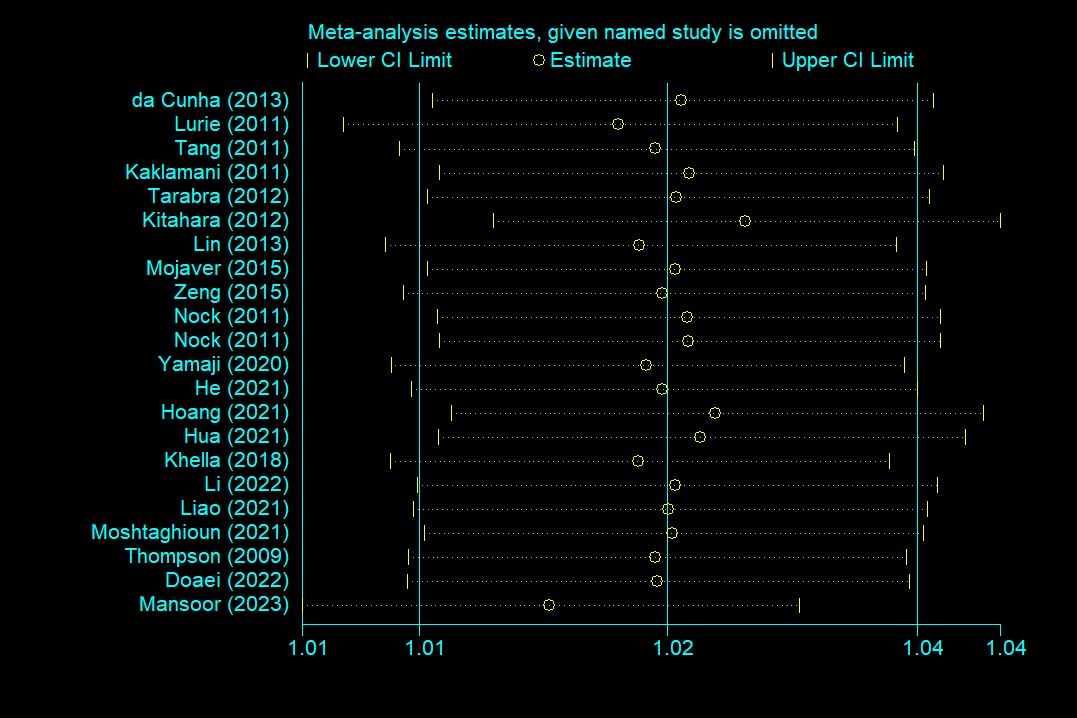

Supplement: Supplementary file 3 — Figure S3. Sensitivity analysis of FTO rs9939609 (allelic comparison B vs. A). [file CNR2-8-e70162-s016.docx]

**Supplementary figure 4.** Sensitivity analysis of *FTO* rs1121980 (allelic comparison B vs. A).


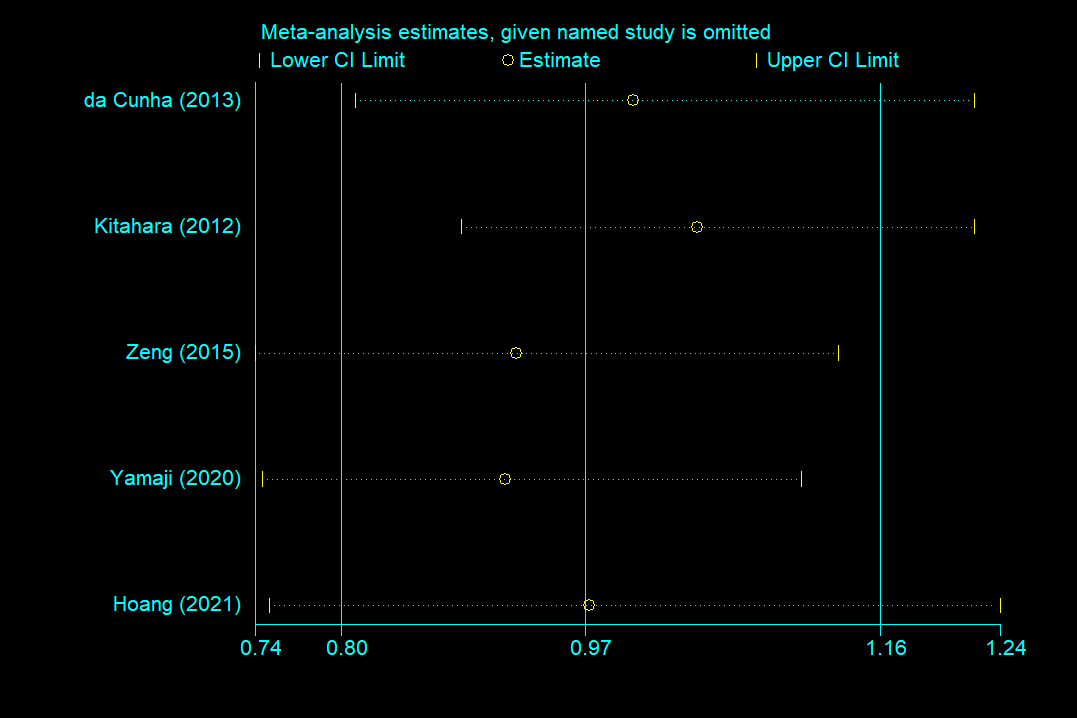

Supplement: Supplementary file 4 — Figure S4. Sensitivity analysis of FTO rs1121980 (allelic comparison B vs. A). [file CNR2-8-e70162-s011.docx]

**Supplementary figure 5.** Sensitivity analysis of *FTO* rs1477196 (allelic comparison B vs. A).


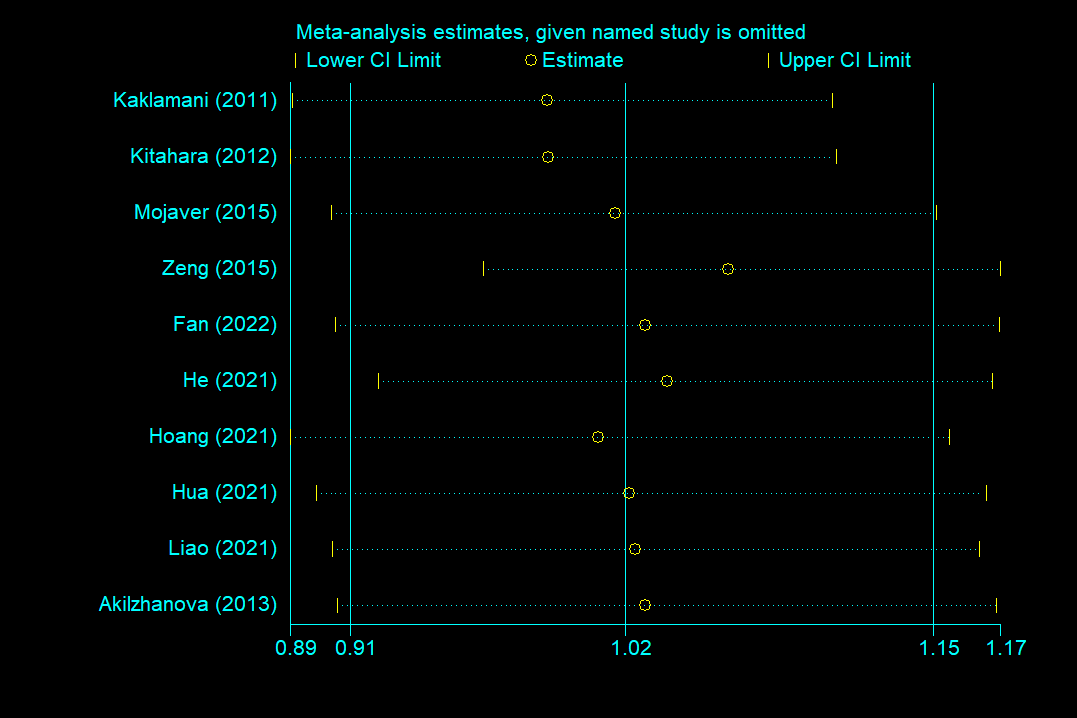

Supplement: Supplementary file 5 — Figure S5. Sensitivity analysis of FTO rs1477196 (allelic comparison B vs. A). [file CNR2-8-e70162-s013.docx]

**Supplementary figure 6.** Sensitivity analysis of *FTO* rs7206790 (allelic comparison B vs. A).


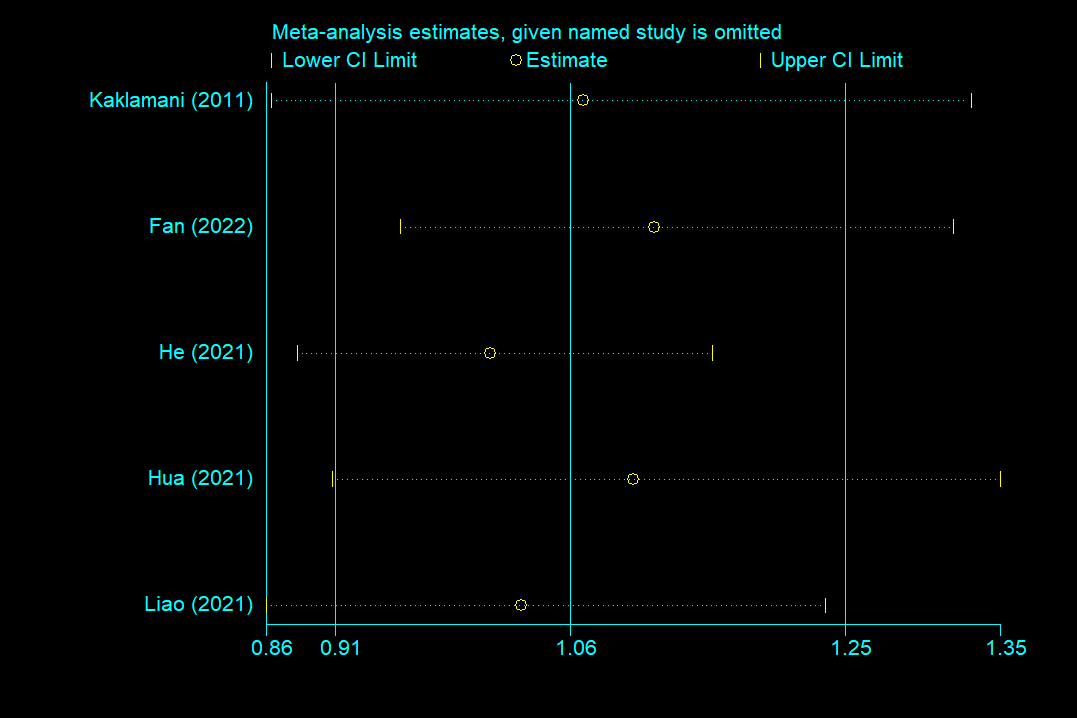

Supplement: Supplementary file 6 — Figure S6. Sensitivity analysis of FTO rs7206790 (allelic comparison B vs. A). [file CNR2-8-e70162-s003.docx]

**Supplementary figure 7.** Sensitivity analysis of *FTO* rs8047395 (allelic comparison B vs. A).


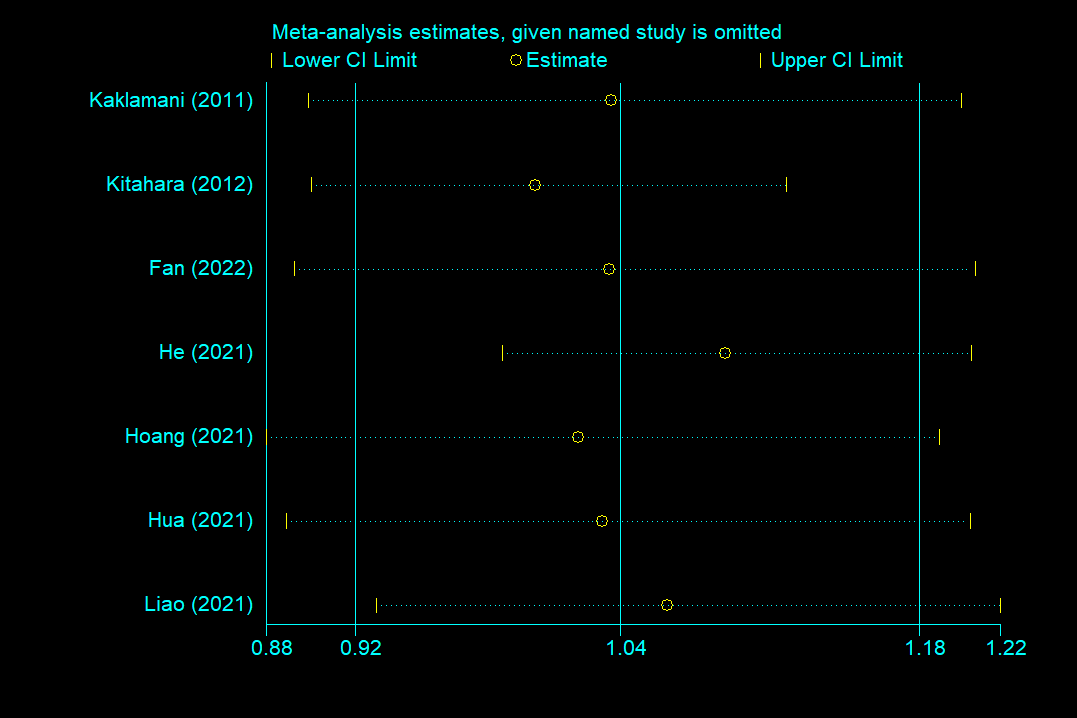

Supplement: Supplementary file 7 — Figure S7. Sensitivity analysis of FTO rs8047395 (allelic comparison B vs. A). [file CNR2-8-e70162-s002.docx]

**Supplementary figure 8.** Sensitivity analysis of *FTO* rs8050136 (allelic comparison B vs. A).


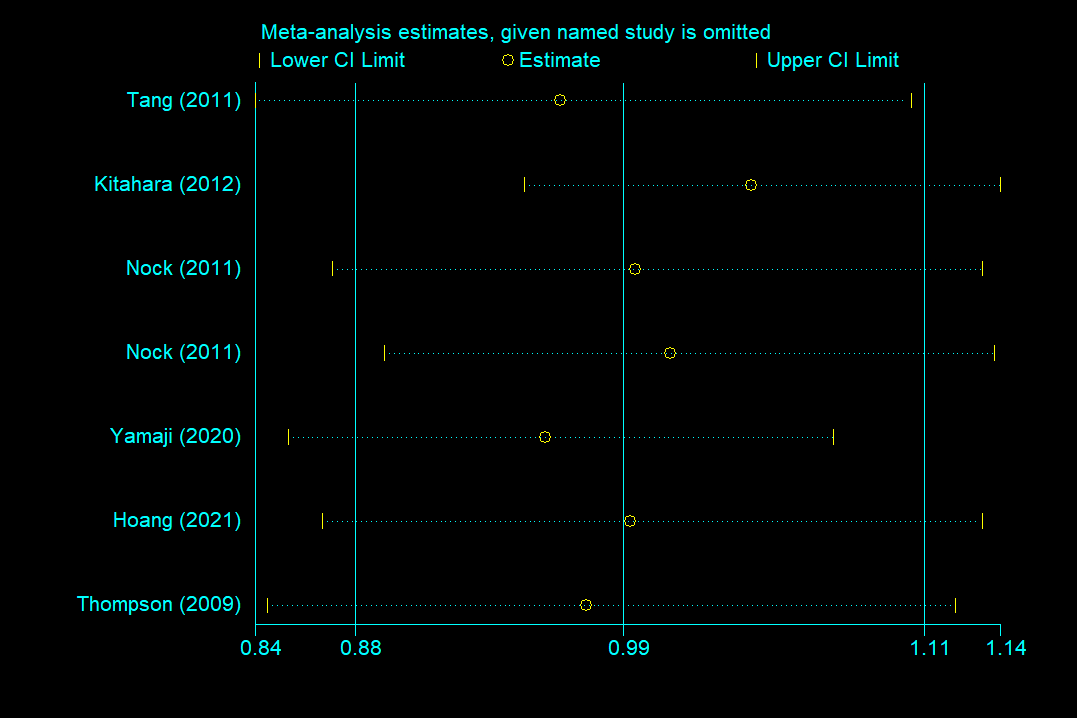

Supplement: Supplementary file 8 — Figure S8. Sensitivity analysis of FTO rs8050136 (allelic comparison B vs. A). [file CNR2-8-e70162-s010.docx]

**Supplementary figure 9.** Funnel plot of *FTO* rs9939609 polymorphism (allelic comparison B vs. A).


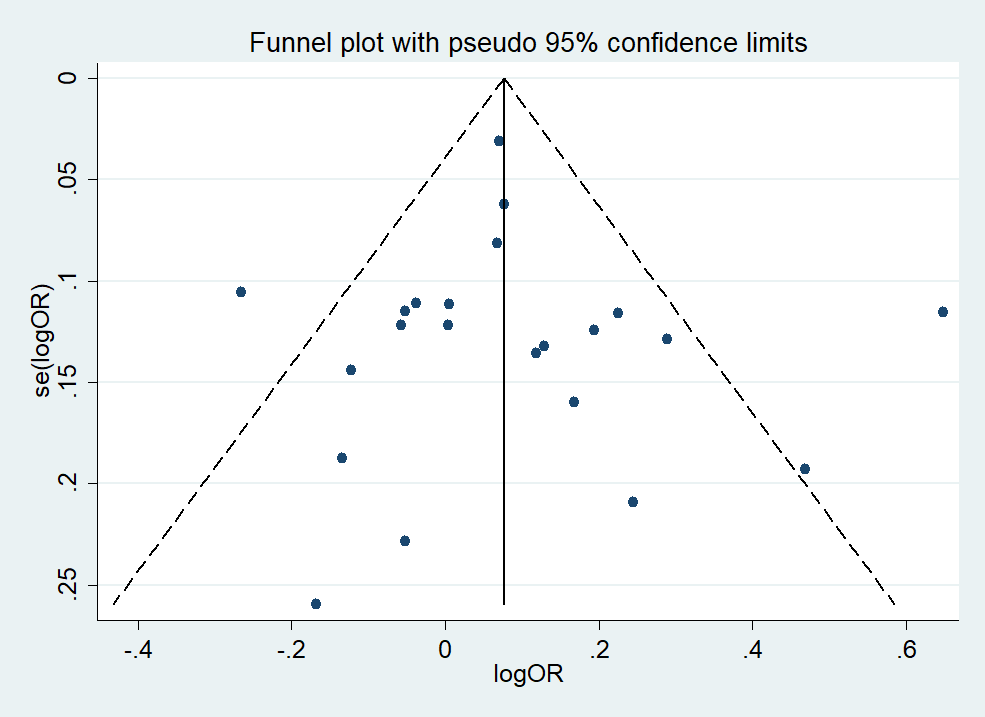

Supplement: Supplementary file 9 — Figure S9. Funnel plot of FTO rs9939609 polymorphism (allelic comparison B vs. A). [file CNR2-8-e70162-s014.docx]

**Supplementary figure 10.** Funnel plot of *FTO* rs1121980 polymorphism (allelic comparison B vs. A).


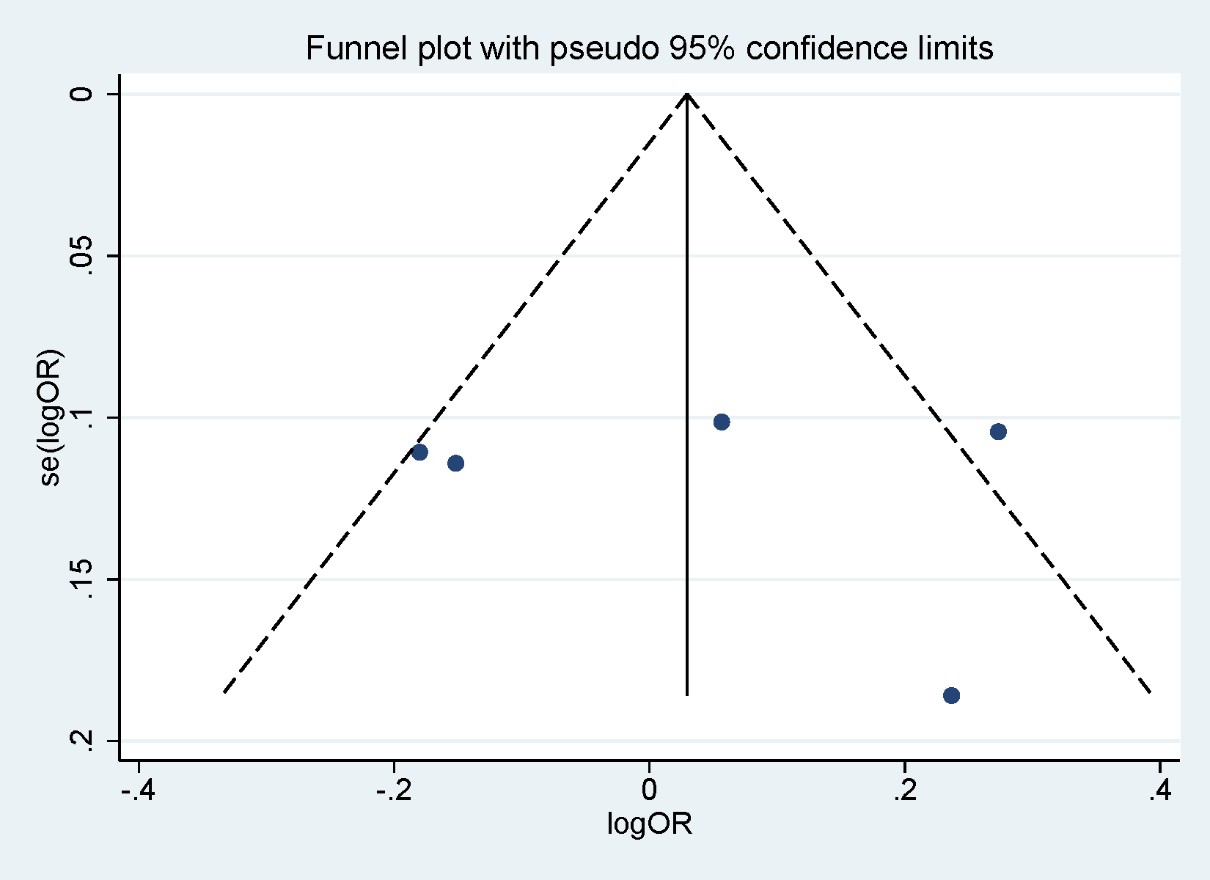

Supplement: Supplementary file 10 — Figure S10. Funnel plot of FTO rs1121980 polymorphism (allelic comparison B vs. A). [file CNR2-8-e70162-s004.docx]

**Supplementary figure 11.** Funnel plot of *FTO* rs1477196 polymorphism (allelic comparison B vs. A).


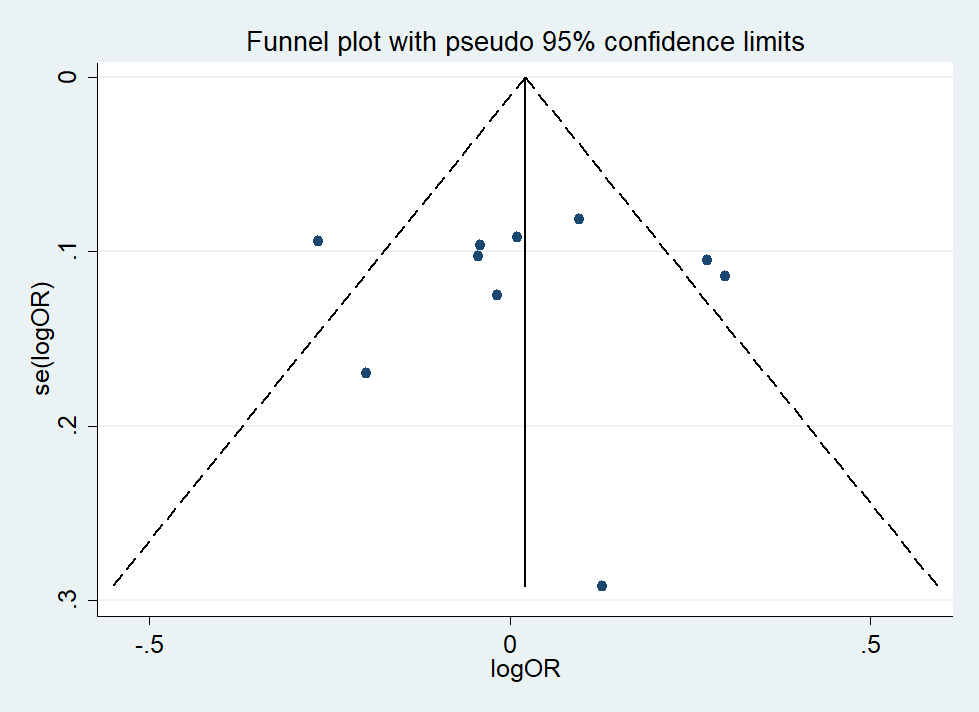

Supplement: Supplementary file 11 — Figure S11. Funnel plot of FTO rs1477196 polymorphism (allelic comparison B vs. A). [file CNR2-8-e70162-s017.docx]

**Supplementary figure 12.** Funnel plot of *FTO* rs7206790 polymorphism (allelic comparison B vs. A).


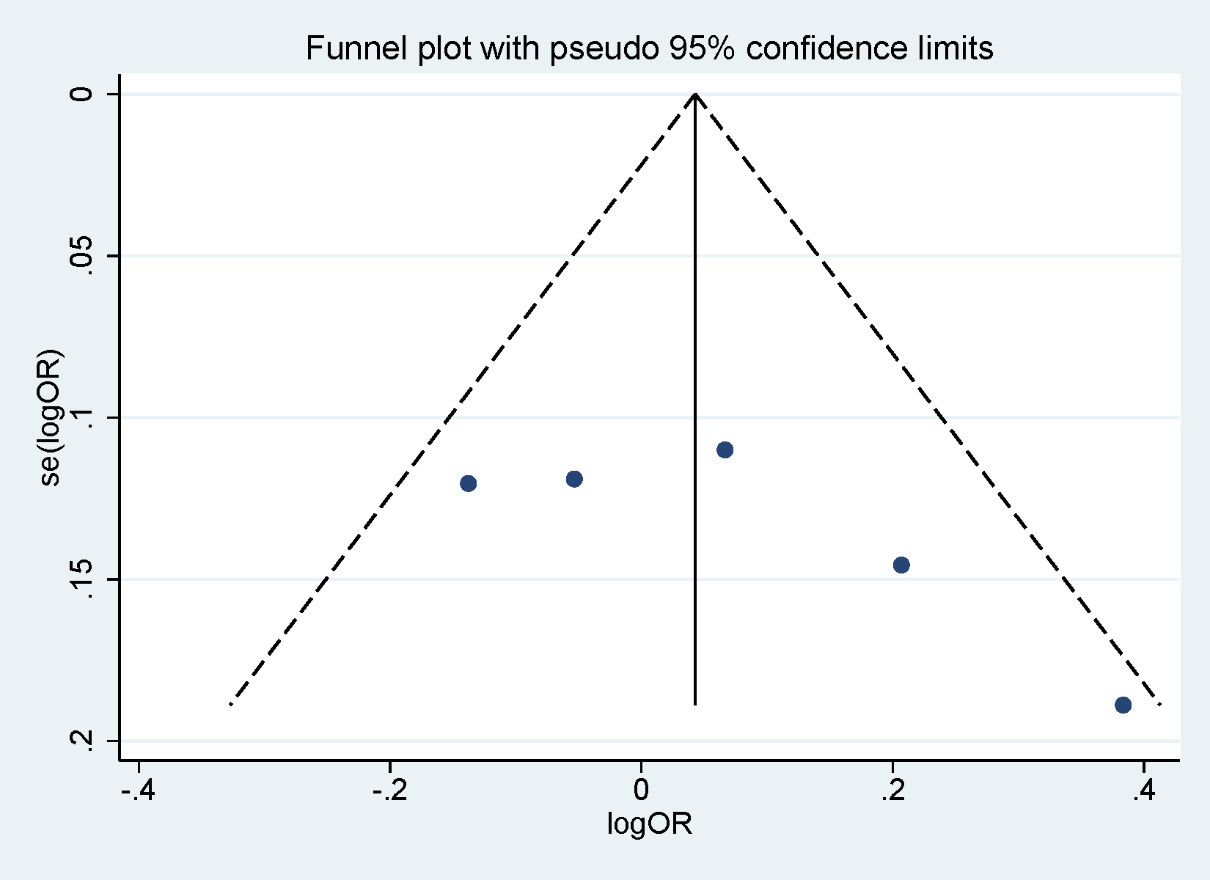

Supplement: Supplementary file 12 — Figure S12. Funnel plot of FTO rs7206790 polymorphism (allelic comparison B vs. A). [file CNR2-8-e70162-s009.docx]

**Supplementary figure 13.** Funnel plot of *FTO* rs8047395 polymorphism (allelic comparison B vs. A).


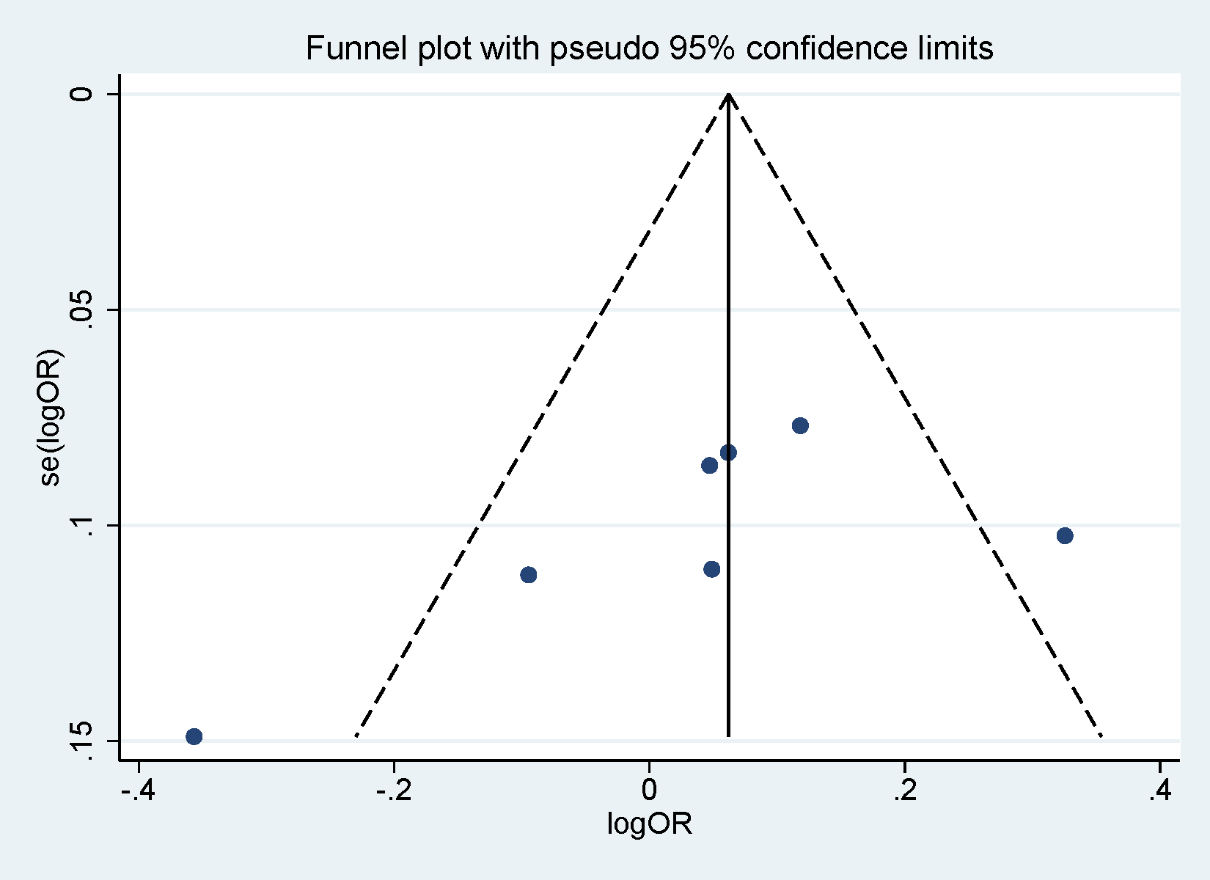

Supplement: Supplementary file 13 — Figure S13. Funnel plot of FTO rs8047395 polymorphism (allelic comparison B vs. A). [file CNR2-8-e70162-s015.docx]

**Supplementary figure 14.** Funnel plot of *FTO* rs8050136 polymorphism (allelic comparison B vs. A).


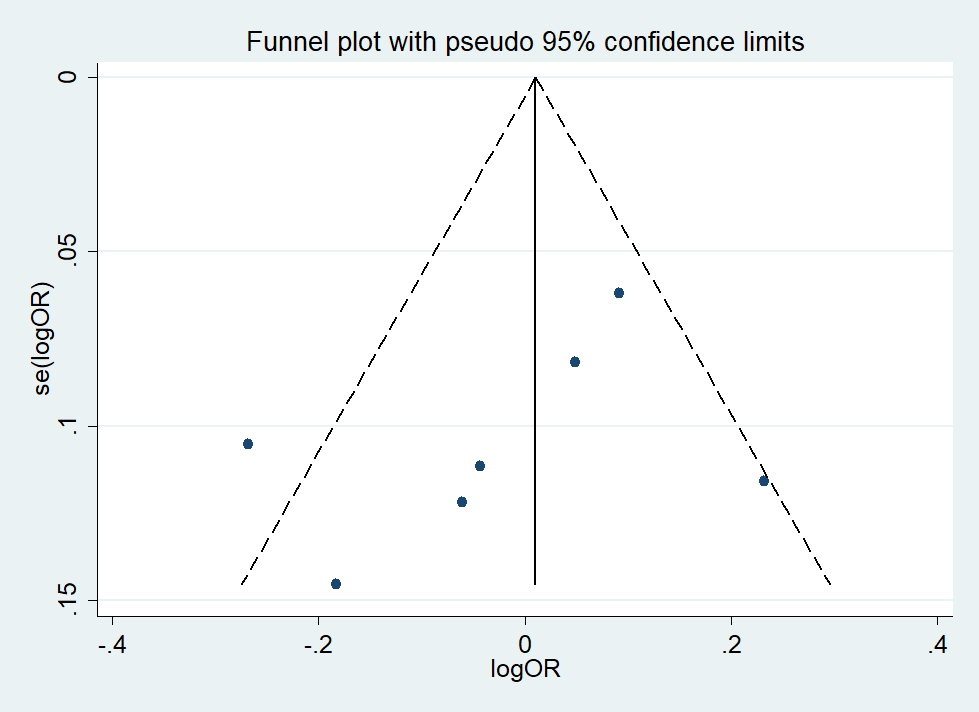

Supplement: Supplementary file 14 — Figure S14. Funnel plot of FTO rs8050136 polymorphism (allelic comparison B vs. A). [file CNR2-8-e70162-s001.docx]
