# Supplementary material for: Polymorphisms in the FTO Gene and Their Association With Cancer Risk: A Comprehensive Review and Meta‐Analysis
Source: Cancer Rep (Hoboken). 2025 May 20;8(5):e70162. doi: 10.1002/cnr2.70162 (PMC12089991; doi:10.1002/cnr2.70162)
Supplement: Supplementary file 15 — Figure S15. Performed an extensive literature review using PubMed, Medline, Scopus, Embase, and Web of Science. [file CNR2-8-e70162-s006.docx]

**Supplementary figure 15.** Performed an extensive literature review using PubMed, Medline, Scopus, Embase, and Web of Science.

**PubMed:** ("FTO"[Mesh] OR "Fat mass and obesity-associated"[tiab]) AND ("polymorphism"[Mesh] OR "mutation"[Mesh] OR "genetic variation"[Mesh] OR "SNP"[tiab]OR "genotype"[Mesh]) AND ("neoplasms"[Mesh] OR "cancer"[tiab] OR "tumor"[tiab] OR "malignancy"[tiab] OR "carcinoma"[tiab] OR "adenocarcinoma"[tiab]) (n=345)

**Medline**: ("FTO" OR "Fat mass and obesity-associated") AND ("polymorphism" OR "mutation" OR "variation" OR "SNP" OR "genotype") AND ("cancer" OR "tumor" OR "neoplasm" OR "malignancy" OR "carcinoma" OR "adenocarcinoma") (n=224)

**Web of Science:** TS=("FTO" OR "Fat mass and obesity-associated") AND

TS=("polymorphism" OR "mutation" OR "variation" OR "SNP" OR "genotype") AND TS=("cancer" OR "tumor" OR "neoplasm" OR "malignancy" OR "carcinoma" OR "adenocarcinoma") (n=249)

Records identified through databases searching

(n=1180)

PubMed:345; Medline:224; Scopus:236; Embase:126; Web of Science:249;

**Scopus :** TITLE-ABS-KEY("FTO" OR "Fat mass and obesity-associated") AND TITLE-ABS-KEY("polymorphism" OR "mutation" OR "variation" OR "SNP" OR "genotype") AND TITLE-ABS-KEY("cancer" OR "tumor" OR "neoplasm" OR "malignancy" OR "carcinoma" OR "adenocarcinoma") (n=236)

**Embase:** ('FTO' OR 'Fat mass and obesity-associated') AND

('polymorphism' OR 'mutation' OR 'variation' OR 'SNP' OR 'genotype') AND

('cancer' OR 'tumor' OR 'neoplasm' OR 'malignancy' OR 'carcinoma' OR 'adenocarcinoma') (n=126)
