# Supplementary material for: Polymorphisms in the FTO Gene and Their Association With Cancer Risk: A Comprehensive Review and Meta‐Analysis
Source: Cancer Rep (Hoboken). 2025 May 20;8(5):e70162. doi: 10.1002/cnr2.70162 (PMC12089991; doi:10.1002/cnr2.70162)
Supplement: Supplementary file 16 — Table S1. Methodological quality of the enrolled studies according to the Newcastle‐Ottawa scale. [file CNR2-8-e70162-s008.docx]

**Supplementary table 1**. Methodological quality of the enrolled studies according to the Newcastle-Ottawa scale.

(continued on next page)

| SNP | First author | Adequacy definition | Representativeness of the cases | Control selection | Control definition | Comparability cases/controls | Exposure ascertainment | Same method ascertainment | Non-response rate |
| --- | --- | --- | --- | --- | --- | --- | --- | --- | --- |
| rs9939609 | da Cunha | * | * | NA | * | ** | * | * | * |
|  | Lurie | * | * | * | * | ** | * | * | * |
|  | Tang | * | * | * | * | ** | * | * | * |
|  | Kaklamani | * | * | * | * | ** | * | * | * |
|  | Tarabra | * | * | * | * | ** | * | * | * |
|  | Kitahara | * | * | * | * | ** | * | * | * |
|  | Lin | * | * | NA | * | ** | * | * | * |
|  | Mojaver | * | * | * | * | ** | * | * | * |
|  | Zeng | * | * | NA | * | ** | * | * | * |
|  | Nock(C) | * | * | * | * | ** | * | * | * |
|  | Nock(A) | * | * | NA | * | ** | * | * | * |
|  | Yamaji | * | * | * | * | ** | * | * | * |
|  | He | * | * | * | * | ** | * | * | * |
|  | Hoang | * | * | * | * | ** | * | * | * |
|  | Hua | * | * | * | * | ** | * | * | * |
|  | Khella | * | * | * | * | ** | * | * | * |
|  | Li | * | * |  | * | ** | * | * | * |
|  | Liao | * | * | * | * | ** | * | * | * |
|  | Moshtaghioun | * | * | * | * | ** | * | * | * |
|  | Thompson | * | * | * | * | ** | * | * | * |
|  | Doaei | * | * | * | * | ** | * | * | * |
|  | Mansoor | * | * | * | * | ** | * | * | * |
|  | Liao | * | * | * | * | ** | * | * | * |
| rs1477196 | Kaklamani | * | * | * | * | ** | * | * | * |
|  | Kitahara | * | * | * | * | ** | * | * | * |
|  | Mojaver | * | * | * | * | ** | * | * | * |
|  | Zeng | * | * | * | * | ** | * | * | * |
|  | Fan | * | * | NA | * | ** | * | * | * |
|  | He | * | * | NA | * | ** | * | * | * |
|  | Hoang | * | * | NA | * | ** | * | * | * |
|  | Hua | * | * | NA | * | ** | * | * | * |
|  | Liao | * | * | NA | * | ** | * | * | * |
|  | Akilzhanova | * | * | * | * | ** | * | * | * |
| rs8047395 | Kaklamani | * | * | * | * | ** | * | * | * |
|  | Kitahara | * | * | * | * | ** | * | * | * |
|  | Fan | * | * | * | * | ** | * | * | * |
|  | He | * | * | * | * | ** | * | * | * |
|  | Hoang | * | * | * | * | ** | * | * | * |
|  | Hua | * | * | * | * | ** | * | * | * |
|  | Liao | * | * | * | * | ** | * | * | * |
| rs7206790 | Kaklamani | * | * | * | * | ** | * | * | * |
|  | Fan | * | * |  | * | ** | * | * | * |
|  | He | * | * | * | * | ** | * | * | * |
|  | Hua | * | * | * | * | ** | * | * | * |
|  | Liao | * | * | * | * | ** | * | * | * |
| rs8050136 | Tang | * | * | * | * | ** | * | * | * |
|  | Kitahara | * | * | * | * | ** | * | * | * |
|  | Nock(C) | * | * | * | * | ** | * | * | * |
|  | Nock(A) | * | * | * | * | ** | * | * | * |
|  | Yamaji | * | * | * | * | ** | * | * | * |
|  | Hoang | * | * | * | * | ** | * | * | * |
|  | Thompson | * | * | * | * | ** | * | * | * |
| rs1121980 | da Cunha | * | * | * | * | ** | * | * | * |
|  | Kitahara | * | * | * | * | ** | * | * | * |
|  | Zeng | * | * | * | * | ** | * | * | * |
|  | Yamaji | * | * | * | * | ** | * | * | * |
|  | Hoang | * | * | * | * | ** | * | * | * |

Note: A study can be awarded a maximum of one star for each numbered item within the Selection and Exposure categories. A maximum of two stars can be given for Comparability. SNP, single nucleic polymorphism; NA, not applicable; C, Caucasian; A, Asian.

.
