# Supplementary material for: Polymorphisms in the FTO Gene and Their Association With Cancer Risk: A Comprehensive Review and Meta‐Analysis
Source: Cancer Rep (Hoboken). 2025 May 20;8(5):e70162. doi: 10.1002/cnr2.70162 (PMC12089991; doi:10.1002/cnr2.70162)
Supplement: Supplementary file 17 — Table S2. Publication bias of the six polymorphisms of FTO. [file CNR2-8-e70162-s007.docx]

**Supplementary table 2**. Publication bias of the six polymorphisms of *FTO*.

| Variables | Allelic contrast (*p*) |  | Dominant model (*p*) |  | Recessive model (*p*) |
| --- | --- | --- | --- | --- | --- |
| rs9939609 |  |  |  |  |  |
| Egger’s test | 0.814 |  | 0.866 |  | 0.430 |
| Begg’s test | 0.573 |  | 0.804 |  | 0.934 |
| rs1477196 |  |  |  |  |  |
| Egger’s test | 0.721 |  | 0.721 |  | 0.210 |
| Begg’s test | 0.567 |  | 0.610 |  | 0.311 |
| rs8047395 |  |  |  |  |  |
| Egger’s test | 0.181 |  | 0.649 |  | **0.013** |
| Begg’s test | 0.072 |  | 0.764 |  | **0.035** |
| rs7206790 |  |  |  |  |  |
| Egger’s test | 0.120 |  | **0.011** |  | 0.335 |
| Begg’s test | 0.462 |  | 0.086 |  | 0.221 |
| rs8050136 |  |  |  |  |  |
| Egger’s test | 0.368 |  | 0.764 |  | 0.548 |
| Begg’s test | 0.277 |  | 0.438 |  | 0.540 |
| rs1121980 |  |  |  |  |  |
| Egger’s test | 0.800 |  | 0.557 |  | 0.382 |
| Begg’s test | 1.000 |  | 0.806 |  | 0.462 |

Abbreviations: NA, not applicable.
